# Family structure as a predictor of screen time among youth

**DOI:** 10.7717/peerj.1048

**Published:** 2015-06-25

**Authors:** Rachel McMillan, Michael McIsaac, Ian Janssen

**Affiliations:** 1 Department of Public Health Sciences, Queen’s University, Kingston, Ontario, Canada; 2 School of Kinesiology and Health Studies, Queen’s University, Kingston, Ontario, Canada

**Keywords:** Adolescent, Health survey, Sedentary behavior, Family, Parents, Health behavior, Television, Computer, Video games

## Abstract

The family plays a central role in the development of health-related behaviors among youth. The objective of this study was to determine whether non-traditional parental structure and shared custody arrangements predict how much time youth spend watching television, using a computer recreationally, and playing video games. Participants were a nationally representative sample of Canadian youth (*N* = 26,068) in grades 6–10 who participated in the 2009/10 Health Behaviour in School-aged Children Survey. Screen time in youth from single parent and reconstituted families, with or without regular visitation with their non-residential parent, was compared to that of youth from traditional dual-parent families. Multiple imputation was used to account for missing data. After multiple imputation, the relative odds of being in the highest television, computer use, video game, and total screen time quartiles were not different in boys and girls from non-traditional families by comparison to boys and girls from traditional dual-parent families. In conclusion, parental structure and child custody arrangements did not have a meaningful impact on screen time among youth.

## Introduction

Sedentary activities, including screen time behaviors such as watching television, using a computer and playing video games, have become ubiquitous in the lives of most young people. In Canada, fewer than 1 in 5 youth aged 10–16 meet the public health recommendation to limit their total recreational screen time to 2 h or less per day (Freeman, King & Pickett, 2012). This is concerning given the findings of a recent systematic review which concluded that excessive screen time in youth is independently associated with several physical, mental, and social health problems such as obesity, the metabolic syndrome, decreased academic achievement, and engagement in antisocial behaviors (Tremblay et al., 2011b). Understanding the determinants of youths’ screen time is therefore a public health priority.

One such determinant of youth screen time may be family structure, which is also associated with a wide range of behavioral, developmental, and health-related outcomes (Bramlett & Blumberg, 2007; Jablonska & Lindberg, 2007). Single parent families and reconstituted families (i.e., families headed by a parent and their partner) are more likely to be of low socioeconomic status than traditional families (Thomson & McLanahan, 2012). Furthermore, single parents may face time constraints that limit their ability to monitor or co-participate in their children’s health-related behaviors (Quarmby, Dagkas & Bridge, 2011). Approximately one in three Canadian youth have a non-traditional family structure (Freeman, King & Pickett, 2012).

Quantitative studies examining the association between family structure and screen time have produced inconsistent results. Some studies report that youth from non-traditional families accumulate more screen time (Lindquist, Reynolds & Goran, 1999; Gorley, Marshall & Biddle, 2004; Quarmby & Dagkas, 2010), while others show that the relationship holds only for girls (Bagley, Salmon & Crawford, 2006; Hesketh, Crawford & Salmon, 2006; Sisson & Broyles, 2012) or boys (Gorely et al., 2009) and still other studies show null results (Salmon et al., 2005; Hardy et al., 2006). A major limitation of these studies is that they did not consider the diversity of modern families. The majority defined parental family structure as simply single- or dual-parent, therefore overlooking potential differences between traditional dual-parent families and reconstituted dual-parent families that include a stepparent or parent’s partner (Thomson & McLanahan, 2012). No studies have looked at how shared custody arrangements, which may involve the youth visiting or living with a non-residential parent, affect screen time. This is of interest given that spending time with a non-residential parent may negate some of the negative health outcomes related to being from a single parent family (Bauserman, 2002; Jablonska & Lindberg, 2007; Bjarnason, Bendtsen & Borup, 2012).

The purpose of this study was to examine whether family structure, determined based on the number of adults in the home and their relationship to the young person, is associated with screen time. This study also considered whether regular contact with a non-residential parent influenced this relationship. Ultimately, it is hoped that this research will contribute to our understanding of how excessive screen time behaviors develop and assist in identifying high-risk youth for targeted interventions.

## Materials and Methods

### Study design and population

Study data are based on the nationally representative cross-sectional 2009/2010 Canadian Health Behaviour in School-aged Children Survey (HBSC). The HBSC is conducted every 4 years in 43 countries in collaboration with the World Health Organization (Currie, Nic Gabhainn & Godeau, 2009; Freeman, King & Pickett, 2012). This study is limited to the Canadian data. The HBSC consists of a standardized self-report survey filled out in a classroom setting, with the goal of determining the prevalence and distribution of a wide range of psychological, social and physical determinants of health in adolescents. All items in the HBSC study are continuously developed, piloted, and validated by the HBSC international network (Currie, Nic Gabhainn & Godeau, 2009; Freeman, King & Pickett, 2012).

Ethics approval for the 2009/2010 Canadian HBSC was given from Health Canada and the General Research Ethics Board at Queen’s University. Depending on the jurisdictional requirements at the participating schools, informed consent from the parents/guardians of participating students could have been obtained in an active or passive manner. In both situations a letter of information was sent home to parents/guardians. If active consent was required, the parent/guardian had to sign the letter and it had to be returned to the school or the student was not allowed to participate. If passive consent was required, the parent did not have to sign or return the letter if they allowed their child to participate. Conversely, they were asked to sign and return the letter if they did not want their child to participate. Irrespective of whether active or passive consent was used, student participants had to provide their informed consent, which was demonstrated by their willingness to complete the survey.

The 2009/2010 Canadian HBSC had a 77% response rate. The final sample consisted of 26,068 students in grades 6–10 (approximate ages 11-15 years) from 436 public schools across Canada. All provinces and territories participated, with the exceptions of New Brunswick and Prince Edward Island. The provincial samples were obtained using a two-tiered cluster-sampling procedure to sample entire classrooms for participation; all students living in the three territories were invited to participate if they met the study inclusion criteria to ensure adequate representation. Students attending private, on-reserve, special needs or home-based schools were excluded, as were those who were absent from school on the day of the survey.

### Exposure (family structure)

Information on family structure was derived from two questions on the HBSC. The first asked participants to check off the adults who live in the home *“where [they] live all or most of the time”* from a list of choices including mother, father, stepmother (or father’s girlfriend) and stepfather (or mother’s boyfriend). The second asked participants to indicate whether they had a second home and, if they did, to identify how often they stayed there (“*half the time*,” “*regularly but less than half the time*,” “*sometimes*” or “*hardly ever*”). Families were defined as traditional (includes both a mother and a father), single parent (includes either a mother or a father), or reconstituted (includes either a mother or a father and either a stepmother/father’s girlfriend or stepfather/mother’s boyfriend). Adolescents from non-traditional families were further defined as having “regular visitation” with a second parent if they had a second home and reported visiting it “*half the time*” or “*regularly but less than half the time*” and “irregular visitation” if the adolescent reported not having a second home, or having a second home but visiting it “*sometimes*” or “*hardly ever*.” Youths who reported that neither their mother nor their father lived in their primary home constituted ∼4% of the sample and were excluded from the analysis.

### Outcome (screen time)

Information on the three screen time behaviors of interest was obtained by asking participants to report how many hours in their free time on a typical weekday and weekend day they usually “watch television (including videos and DVDs),” “play games on a computer or games console (Playstation, Xbox, Gamecube, etc.)” or “use a computer for chatting on-line, internet, emailing, homework, etc.” For each question there were 9 ordinal response options, ranging from “none at all” to “about 7 or more hours a day.” A weighted average of weekly time spent in each screen time behavior was calculated. A validation study of a similar questionnaire measuring weekly television use in adolescents showed that participants’ responses were significantly correlated (*r* = 0.47) with television viewing time as measured using a detailed 7-day log (Schmitz et al., 2004). Screen time was dichotomized so that those in the highest quartile of weekly time spent in each screen time behavior could be compared to those in the lower three quartiles. Separate analyses were performed comparing those who spent >2 h in each screen time behavior to those who spent ≤2 h, as screen time guidelines have recommended this threshold (American Academy of Pediatrics, 2001; Tremblay et al., 2011a).

### Covariates

Potential covariates for the model of the relationship between family structure and screen time were selected based on previous literature and their availability within the HBSC. These included gender, grade, ethnicity (Canadian, which includes those who self-identify as both Caucasian and Aboriginal ethnicity, East and Southeast Asian, South Asian, Black, Arab, or other, which includes those of mixed ethnicity and who also who self-identify as other), immigration status (non-immigrant/immigrated >5 years ago or immigrated ≤5 years ago), presence of siblings (yes or no), and family affluence. Family affluence was measured through a self-report item on the HBSC that asked students to report “how well off [they] think [their] family is,” with five ordinal responses ranging from “not at all well off” to “very well off.” The top and bottom two responses were combined to create a three-level ordinal measure of perceived family affluence.

### Statistical analysis

All analyses used survey procedures in SAS 9.4 to account for the complex sampling design used by the HBSC, including clustering at the classroom and provincial levels and sampling weights. All analyses were stratified by gender. The HBSC sample population was characterized using simple descriptive statistics. Cross-tabulations of the average amount of time spent in each screen time behavior by family structure were calculated, and differences between family structures were assessed using analysis of covariance after adjusting for relevant confounders. A Bonferroni correction was used to account for 10 multiple group comparisons. Note that the 95% confidence intervals (CI) for the group means that are presented in the manuscript were not adjusted to account for multiple group comparisons; we only corrected the *P* values used to denote statistical group differences.

Multiple logistic regression was used to further quantify associations between family structure and screen time behavior. Final covariate selection for the multivariate models was performed through backwards deletion using a 10% change-in-estimate threshold (Rothman & Greenland, 1998). If a covariate significantly changed the regression coefficient of at least one non-traditional family structure in at least one of the models, it was included in all final models. All covariates of interest met this criterion.

Approximately 15% of participants were missing data for at least one relevant exposure, outcome, or confounding variable. Because we were concerned that this would bias our results, we performed two forms of multiple imputation for the missing data: one based on fully conditional specification and the other based on the use of Monte Carlo Markov Chains. Because the two imputation methods produced consistent and similar results, only the results from the fully conditional specification-based imputation are shown in the manuscript. This method is more appropriate for the imputation of categorical data because it does not assume normality of imputed variables (Lee & Carlin, 2010). In both imputation procedures, all variables were included for the multiple imputation, and 50 concatenated datasets were created rather than the typical 5 in an effort to reduce any potential bias caused by rounding.

## Results

### Sample characteristics

Demographic characteristics of the sample are in Table 1. The majority of participants had lived in Canada for more than 5 years (94.4%), were of Canadian (Caucasian or Aboriginal) ethnicity (75.3%), and considered themselves to be of higher than average socioeconomic status (53.7%). Most participants lived in traditional, dual-parent families (65.0%), followed by single parent families with irregular visitation with their non-custodial parent (13.4%), reconstituted families with irregular visitation (6.6%), single parent families with regular visitation (4.2%) and finally reconstituted families with regular visitation (2.7%). Most participants exceeded Canada’s recreational screen time guidelines, with 80.6% reporting that they spent a cumulative total of more than 2 h per day watching television, playing video games, and using a computer. The average weekly screen time was 59.4 h in boys and 53.4 h in girls.

**Table 1:** Characteristics of the 2009/10 Canadian health behaviour in school-aged children survey.

Characteristic	*N*	% (95% CI)^*^
**Gender**		
Male	12,878	49.1 (47.7–50.6)
Female	13,169	50.8 (49.4–52.3)
*Missing*	31	0.0 (0.0–0.1)
**Grade**		
Grade 5	55	0.25 (0.00–0.51)
Grade 6	5,110	19.6 (16.1–23.0)
Grade 7	5,205	20.0 (17.6–22.3)
Grade 8	5,266	20.2 (17.7–22.7)
Grade 9	5,395	20.7 (17.4–23.9)
Grade 10	4,871	18.8 (15.5–22.1)
Grade 11	176	0.55 (0.37–0.73)
*Missing*	0	0
**Self-perceived family wealth**		
Low	2,411	9.0 (8.4–9.6)
Average	8,581	31.7 (30.4–33.0)
High	13,466	53.7 (52.1–55.2)
*Missing*	1,620	5.6 (4.6–6.6)
**Immigrant status**		
Lived in Canada ≥5 years	24,709	94.4 (93.4–95.3)
Lived in Canada <5 years	1,093	4.6 (3.8–5.5)
*Missing*	276	1.0 (0.80–1.2)
**Parental structure**		
Traditional family	16,504	65.0 (63.6–66.5)
Single parent with regular visitation	997	4.2 (3.8–4.6)
Single parent with irregular visitation	3,594	13.4 (12.6–14.2)
Reconstituted with regular visitation	662	2.7 (2.4–3.0)
Reconstituted with irregular visitation	1,744	6.6 (6.0–7.1)
Other	1,533	5.0 (4.5–5.5)
*Missing*	1,044	3.0 (2.6–3.5)
**Siblings**		
Only child	3,787	13.5 (12.7–14.3)
≥1 sibling	21,253	83.2 (82.4–84.1)
*Missing*	1,038	3.3 (2.8–3.7)
**Ethnicity**		
Canadian	20,624	75.3 (71.8–78.7)
East and Southeast Asian	1,285	5.7 (4.0–7.3)
South Asian	656	3.2 (2.2–4.2)
Black	481	2.6 (1.9–3.3)
Arab	229	1.3 (0.7–1.8)
Latin American	191	0.9 (0.6–1.2)
Other	2,294	10.0 (9.0–11.0)
*Missing*	318	1.1 (0.9–1.3)
**Television viewing**		
≤2 h/day	12,508	50.5 (49.0–51.9)
>2 h/day	11,128	41.5 (40.0–43.1)
*Missing*	2,442	8.0 (7.1–8.9)
**Video game use**		
≤2 h/day	15,512	32.1 (30.9–33.2)
>2 h/day	8,170	60.2 (58.7–61.8)
*Missing*	2,396	7.7 (6.8–8.6)
**Computer use**		
≤2 h/day	8,902	36.8 (35.3–38.3)
>2 h/day	14,819	55.5 (53.8–57.2)
*Missing*	2,357	7.7 (6.8–8.5)
**Total screen time**		
≤2 h/day	2,706	9.7 (8.8–10.5)
>2 h/day	20,510	80.6 (79.6–81.7)
*Missing*	2 862	9.7 (8.7–10.6)

**Notes.**

*N* Number of sampled individuals with complete valid data for all variables presented

* Estimated population characteristics after adjusting for sampling weights and clustering.

### Relationships between family structure and screen time

As shown in Table 2, youth from non-traditional families had slightly higher screen time values than youth from traditional families, although few differences were statistically significant and even the significant differences were small in magnitude (e.g., <5 h/week difference between the mean screen time in the non-traditional and traditional).

**Table 2:** Mean weekly hours of screen time (television, video games and computer) according to family structure.

Family structure group	*N*	Television	Computer	Video games	Total screen time
		Mean (95% CI)	Mean (95% CI)	Mean (95% CI)	Mean (95% CI)
***Boys***					
Traditional	8,699	20.2 (19.1–21.3)	15.1 (14.1–16.2)	16.8 (15.8–17.8)	52.1 (49.4–54.9)
Reconstituted with irregular visitation	784	21.7 (19.9–23.4)	16.9 (15.1–18.6)	18.6 (16.9–20.3)	57.1 (53.1–61.2)
Reconstituted with regular visitation	295	19.0 (16.5–21.5)	15.3 (13.0–17.6)	18.7 (16.3–21.1)	53.0 (47.6–58.4)
Single parent with irregular visitation	1,763	20.8 (19.4–22.1)	16.5 (15.2–17.8)	19.5 (18.2–20.7)^*^	56.8 (53.6–59.9)^*^
Single parent with regular visitation	494	21.8 (19.6–23.9)	17.2 (15.1–19.4)	20.3 (18.2–22.3)^*^	59.3 (54.0–64.6)^*^
***Girls***					
Traditional	8,774	19.0 (17.9–20.0)	17.6 (16.6–18.6)	9.7 (8.8–10.7)	46.3 (43.8–48.7)
Reconstituted with irregular visitation	988	20.1 (18.4–21.7)	18.4 (16.9–19.9)	10.4 (8.9–11.8)	48.8 (45.2–52.3)
Reconstituted with regular visitation	414	20.3 (18.2–22.4)	20.4 (18.2–22.6)^*^	11.2 (9.2–13.2)	51.9 (46.6–57.1)
Single parent with irregular visitation	1,860	20.7 (19.5–21.9)^*^	19.4 (18.3–20.5)^*^	11.0 (9.8–12.2)	51.1 (48.2–53.9)^*^
Single parent with regular visitation	581	19.5 (17.8–21.3)	19.2 (17.2–21.2)	11.2 (9.3–13.0)	49.9 (45.5–54.2)

**Notes.**

Data presented as mean (95% confidence interval) after adjusting for sample weights and clustering and the following covariates: grade, immigration, siblings, ethnicity, and family wealth.

* Significantly different from traditional families after taking into account multiple comparisons (Bonferroni-adjusted *p*-value <0.05).

Tables 3 to 6 show odds ratios for being in the highest quartile of weekly screen time by family structure, with traditional families serving as the referent group. Statistically significant (*p* < 0.05, after Bonferroni correction) relationships are shown in bold. There were several significant relationships in the bivariate analyses. However, these significant relationships were weak in strength (i.e., odds ratios <1.25) and were not consistent across gender or the three screen time behaviors. Furthermore, these relationships were no longer significant after controlling for the confounding variables.

**Table 3:** Relationships between family structure and being in the highest television viewing quartile.

Family structure group	Frequency, % (95% CI)	Bivariate analysis, OR (95% CI)	Fully adjusted model,^*^ OR (95% CI)
***Boys***			
Traditional	27.5 (25.5–29.5)	1.00	1.00
Reconstituted with irregular visitation	32.9 (27.8–38.0)	1.21 (0.99–1.49)	1.20 (0.98–1.49)
Reconstituted with regular visitation	20.6 (13.9–27.3)	**0.64 (0.46–0.89)**	**0.66 (0.47–0.92)**
Single parent with irregular visitation	31.9 (28.4–35.5)	1.16 (0.99–1.36)	1.11 (0.94–1.31)
Single parent with regular visitation	32.3 (25.7–38.9)	1.18 (0.91–1.53)	1.19 (0.91–1.55)
***Girls***			
Traditional	24.6 (22.8–26.4)	1.00	1.00
Reconstituted with irregular visitation	28.4 (24.1–32.6)	1.05 (0.88–1.26)	1.05 (0.88–1.26)
Reconstituted with regular visitation	28.6 (21.9–35.4)	1.07 (0.82–1.40)	1.12 (0.86–1.47)
Single parent with irregular visitation	31.3 (28.4–34.2)	**1.21 (1.05–1.40)**	1.14 (0.98–1.32)
Single parent with regular visitation	24.0 (18.9–29.1)	0.84 (0.66–1.07)	0.84 (0.66–1.06)

**Notes.**

All analyses account for sample weights and clustering. Statistically significant odds ratios are shown in bold.

* Adjusted for the following covariates: grade, immigration, siblings, ethnicity, and family wealth.

**Table 4:** Relationships between family structure and being in the highest computer use quartile.

Family structure group	Frequency, % (95% CI)	Bivariate analysis, OR (95% CI)	Fully adjusted model,^*^ OR (95% CI)
***Boys***			
Traditional	20.3 (18.6–22.1)	1.00	1.00
Reconstituted with irregular visitation	26.4 (21.3–31.6)	1.12 (0.90–1.39)	1.07 (0.86–1.35)
Reconstituted with regular visitation	22.9 (15.6–30.2)	0.92 (0.66–1.29)	0.95 (0.68–1.33)
Single parent with irregular visitation	27.3 (24.1–30.5)	1.17 (0.98–1.38)	1.08 (0.91–1.29)
Single parent with regular visitation	25.2 (18.9–31.6)	1.05 (0.79–1.38)	1.14 (0.86–1.50)
***Girls***			
Traditional	27.5 (25.6–29.5)	1.00	1.00
Reconstituted with irregular visitation	33.4 (29.3–37.6)	1.08 (0.92–1.28)	1.02 (0.86–1.21)
Reconstituted with regular visitation	33.0 (26.6–39.5)	1.06 (0.85–1.34)	1.14 (0.90–1.45)
Single parent with irregular visitation	35.1 (31.5–38.8)	**1.17 (1.02–1.34)**	1.10 (0.95–1.26)
Single parent with regular visitation	29.6 (23.3–35.9)	0.91 (0.72–1.15)	0.93 (0.73–1.18)

**Notes.**

All analyses account for sample weights and clustering. Statistically significant odds ratios are shown in bold.

* Adjusted for the following covariates: grade, immigration, siblings, ethnicity, and family wealth.

**Table 5:** Relationships between family structure and being in the highest video game use quartile.

Family structure group	Frequency, % (95% CI)	Bivariate analysis, OR (95% CI)	Fully adjusted model,^*^ OR (95% CI)
***Boys***			
Traditional	33.1 (31.4–34.9)	1.00	1.00
Reconstituted with irregular visitation	39.1 (33.8–44.5)	1.00 (0.82–1.21)	0.97 (0.80–1.18)
Reconstituted with regular visitation	38.2 (30.1–46.3)	0.96 (0.73–1.26)	0.97 (0.74–1.28)
Single parent with irregular visitation	42.4 (39.1–45.6)	1.14 (0.97–1.34)	1.11 (0.94–1.31)
Single parent with regular visitation	43.4 (35.6–51.1)	1.19 (0.93–1.53)	1.18 (0.92–1.52)
***Girls***			
Traditional	15.1 (13.8–16.4)	1.00	1.00
Reconstituted with irregular visitation	16.6 (13.3–20.0)	0.95 (0.77–1.16)	0.92 (0.75–1.13)
Reconstituted with regular visitation	18.3 (13.2–23.4)	1.07 (0.80–1.41)	1.12 (0.84–1.49)
Single parent with irregular visitation	19.1 (16.2–22.0)	1.12 (0.94–1.34)	1.05 (0.88–1.26)
Single parent with regular visitation	17.9 (12.7–23.2)	1.04 (0.79–1.37)	1.06 (0.80–1.39)

**Notes.**

All analyses account for sample weights and clustering. Statistically significant odds ratios are shown in bold.

* Adjusted for the following covariates: grade, immigration, siblings, ethnicity, and family wealth.

**Table 6:** Relationships between family structure and being in the highest total screen time quartile.

Family structure group	Frequency, % (95% CI)	Bivariate analysis, OR (95% CI)	Fully adjusted model,^*^ OR (95% CI)
***Boys***			
Traditional	28.4 (26.3–30.6)	1.00	1.00
Reconstituted with irregular visitation	34.8 (29.6–40.0)	1.07 (0.88–1.30)	1.05 (0.86–1.28)
Reconstituted with regular visitation	29.2 (21.8–36.7)	0.83 (0.62–1.12)	0.85 (0.63–1.15)
Single parent with irregular visitation	37.8 (34.2–41.3)	**1.22 (1.04–1.44)**	1.15 (0.98–1.36)
Single parent with regular visitation	36.4 (29.3–43.5)	1.15 (0.90–1.47)	1.19 (0.92–1.52)
***Girls***			
Traditional	21.6 (19.8–23.4)	1.00	1.00
Reconstituted with irregular visitation	24.0 (19.8–28.1)	0.96 (0.81–1.15)	0.93 (0.78–1.11)
Reconstituted with regular visitation	26.7 (20.5–32.9)	1.11 (0.86–1.43)	1.19 (0.91–1.54)
Single parent with irregular visitation	27.7 (24.6–30.8)	**1.17 (1.00–1.36)**	1.08 (0.93–1.27)
Single parent with regular visitation	23.7 (18.2–29.3)	0.95 (0.74–1.22)	0.96 (0.75–1.23)

**Notes.**

All analyses account for sample weights and clustering. Statistically significant odds ratios are shown in bold.

* Adjusted for the following covariates: grade, immigration, siblings, ethnicity, and family wealth.

The associations between family structure and screen time did not change in a meaningful way when the definition of elevated screen time was changed from being in the highest quartile of screen usage to exceeding a 2 h/day threshold (data not shown). The associations also did not change based on the choice of imputation methods (data not shown). The relationships observed in the imputed data were, however, different from those estimated using a full case analysis in which observations with missing data were simply deleted. For example, the odds ratios for boys from single parent families being in the highest quartile of video game usage were significant in the full case analysis (OR 1.40, 95% CI [1.22–1.60] for those with irregular visitation and OR 1.48, 95% CI [1.05–2.09] for those with regular visitation), indicating that boys from single parent families watched more television than those from traditional families regardless of visitation. However, these odds ratios were not significant when using the imputation models to reduce biases resulting from missing data (OR 1.13, 95% CI [0.96–1.34] and OR 1.17, 95% CI [0.91–1.51], respectively).

## Discussion

The goal of this study was to examine non-traditional family structures—specifically single parent or reconstituted families, as well as shared custody arrangements—as potential predictors of excessive screen time in youth. While youth from non-traditional families did spend slightly more hours per week in total screen time, these differences were subtle (<15%) and generally not statistically significant. Youth from non-traditional families were also not significantly more likely to exceed screen time guidelines or be in the highest quartile of weekly television, video game, or computer use after controlling for relevant covariates.

Our findings were consistent with previous quantitative studies of television viewing in youth, which have generally shown null results or subtle or insignificant increases in television-viewing in youth from single parent families (Lindquist, Reynolds & Goran, 1999; Salmon et al., 2005; Bagley, Salmon & Crawford, 2006; Hesketh, Crawford & Salmon, 2006; Gorely et al., 2009) and youth from reconstituted families (Quarmby, Dagkas & Bridge, 2011; Sisson & Broyles, 2012). Only one previous study has looked at the influence of family structure on screen behaviors other than television (Gorely et al., 2009). While we did observe descriptive differences in the amount of time spent playing video games and using a computer by gender, we did not observe any meaningful interactions between gender and family structure.

To our knowledge, this study was the first to consider visitation with the non-residential parent as a potential predictor of differences in screen time behavior. It has been suggested that youth who regularly travel to visit another parent may have less time to do anything but sedentary activities (Quarmby & Dagkas, 2010). Our research did not support this argument, showing no consistent significant differences in screen time based on visitation with the non-custodial parent. This is consistent with evidence demonstrating that screen time is associated with mental and emotional health (Tremblay et al., 2011b), and additional findings that children in the shared physical custody of their separated parents experience similar emotional well-being to those who live in a traditional family (Bauserman, 2002; Jablonska & Lindberg, 2007; Bjarnason, Bendtsen & Borup, 2012).

It has been hypothesized that time and energy constraints experienced by single parents may create environments that encourage sedentary behavior in their children (Quarmby & Dagkas, 2010; Quarmby, Dagkas & Bridge, 2011). Our research does not support that argument. Screen time, and particularly time spent watching television, was high in all family structure groups and not just among youth from single parent homes. Therefore, while interventions to decrease screen time in youth are necessary, these interventions likely do not need to target youth in non-traditional family structures as a high risk group.

A strength of this study is its large sample size, which allowed us to delineate diverse family structures in our analyses. Furthermore, the findings are generalizable as they are based on a nationally representative sample. Another strength was the use of multiple imputation to handle missing data in partially completed surveys, which is a common concern when analyzing survey data (Lee & Carlin, 2010). In our study ∼15% of observations had data missing for at least one variable. Multiple imputation avoids the assumption that the associations observed between screen time and family structure are the same among those who answered all questions and those who did not. The fact that the results from the imputed analyses were somewhat different from those based on full cases suggests that we would have biased some of the effect estimates had we not used imputation. This has implications for future studies with HBSC and other youth survey data.

Our study also had important weaknesses. Several potential covariates and mediators were not available in the dataset, such as family co-participation in screen time behaviors, parental employment status, parental modeling, and house rules related to screen time. We were also unable to determine how long participants had been in their current family structure. All of the variables used were based on self-report, and were therefore subject to recall and/or social desirability bias. Finally, selection bias was a concern given that youth who did not provide consent or who were absent from school on the day of the survey may have been systematically different from those who did participate.

In conclusion, family structure was not a meaningful predictor of screen time in this large and representative sample of Canadian youth. Future research should focus on identifying further determinants of sedentary behavior, and the mechanisms through which family structure influences other health behaviors and outcomes.

## Additional Information and Declarations

### Data Deposition

The following information was supplied regarding the deposition of related data: Requests for access to the Canadian Health Behaviour in School-Aged Children study data can be made to the Social Program Evaluation Group at Queen’s University at the following email address: spegmail@queensu.ca.
